# Effects of Piperine on the Intestinal Permeability and Pharmacokinetics of Linarin in Rats

**DOI:** 10.3390/molecules19055624

**Published:** 2014-04-30

**Authors:** Xinchi Feng, Youping Liu, Xin Wang, Xin Di

**Affiliations:** School of Pharmacy, Shenyang Pharmaceutical University, 103 Wenhua Road, Shenyang 110016, China; E-Mails: fengxinchi19880211@163.com (X.F.); yp-liu@163.com (Y.L.); wangxin68k@163.com (X.W.)

**Keywords:** linarin, piperine, P-glycoprotein, intestinal permeability, pharmacokinetics

## Abstract

Although linarin possesses diverse pharmacological activities, its poor oral bioavailability has been a concern for further development. The present study aimed to demonstrate the feasibility of improving the oral absorption of linarin in rats with a bioenhancer‒piperine. First, the intestinal permeability of linarin in the presence and absence of verapamil or piperine was investigated using an *in situ* single-pass rat intestinal perfusion method. A significant increase in the *P_eff_* when co-perfused with verapamil or piperine indicated that piperine effectively inhibited P-glycoprotein mediated efflux of linarin. Then, the pharmacokinetic profiles of linarin in rats after oral administration of linarin (50 mg/kg) alone and in combination with piperine (20 mg/kg) were determined using a validated LC–MS/MS method. The results showed that piperine increased the plasma exposure (AUC) of linarin by 381% along with an increase in the C_max_ by 346% and the T_max_ from 0.05 h to 0.2 h. The present study revealed that piperine significantly enhanced the oral absorption of linarin in rats by inhibiting P-glycoprotein mediated cellular efflux during the intestinal absorption and likely simultaneously by inhibiting the metabolism of linarin.

## 1. Introduction

**Figure 1:**
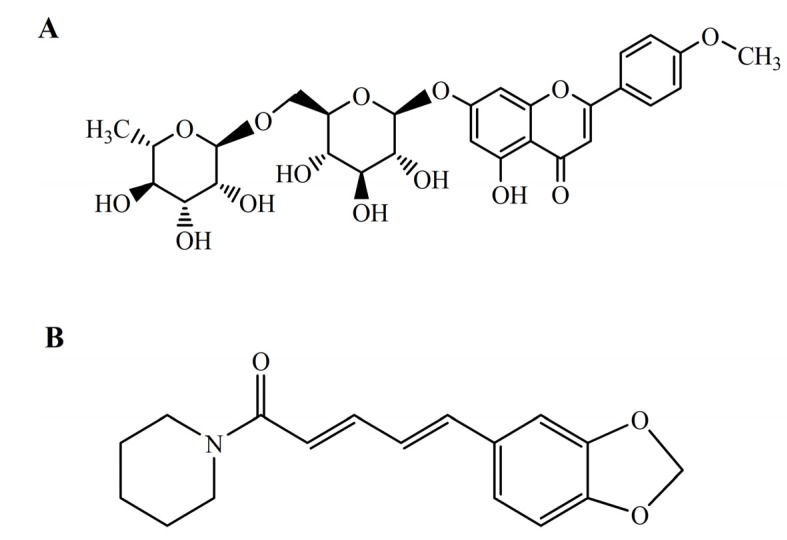
Chemical structures of linarin (**A**) and piperine (**B**).

Linarin (acacetin-7-*O*-β-D-rutinoside, Figure 1A) is a natural flavonoid glycoside commonly found in many herbal plants like *Flos chrysanthemi indici*, *Buddleja officinalis*, *Cirsium setosum*, *Mentha arvensis* and *Buddleja davidii*. It has previously been reported to possess analgesic, antipyretic and anti-inflammatory activities [1,2]. Recent research has revealed that linarin is also a strong acetylcholinesterase inhibitor and exerts robust neuroprotective effects [3,4,5,6]. The diverse pharmacological activities of linarin suggested that linarin might be a promising drug candidate for the treatment of various diseases. However, the poor oral bioavailability of linarin has been a concern as it may hinder further development and clinical application of linarin.

In fact, low bioavailability is a common feature of flavonoids. Many attempts have been made to increase the bioavailability of flavonoids, including improving the intestinal absorption, enhancing metabolic stability and developing novel delivery systems [7]. Interestingly, some bioactive compounds of natural origin (such as piperine, allicin, genistein, *etc.*) have been shown to possess bioavailability enhancing activity, accordingly they are called bioenhancers [8]. Piperine (Figure 1B), a main active component in both black pepper (*Piper nigrum* Linn.) and long pepper (*Piper longum* Linn.), was validated as the world’s first bioenhancer in 1979 [9]. The possible mechanisms of bioavailability enhancement action of piperine include increasing blood supply to the gastrointestinal tract, decreasing gastrointestinal emptying, and inhibiting drug metabolizing enzymes and P-glycoprotein (P-gp) [10,11,12,13]. It has been demonstrated to be effective in enhancing the bioavailability of rifampicin, phenytoin, sulfadiazine, resveratrol, fexofenadine, tetracycline, propranolol and theophylline, *etc.* [14,15,16,17,18]. In particular, a dual drug-loaded nanoformulation containing curcumin [which has demonstrated efficacy as an anticancer agent but has a very low bioavailability (about 1%)] and piperine for the treatment of multidrug-resistant cancers has been recently proposed and prepared to enhance the bioavailability of curcumin [19].

The aim of the present study was to demonstrate the feasibility of improving the oral absorption of linarin in rats with piperine. First, an *in situ* model, the single-pass perfused rat intestine, was chosen as an experimental system to assess the modulation of intestinal absorption of linarin by piperine. Then, comparative pharmacokinetic study of linarin in rats with and without co-administration of piperine was conducted to further confirm the effect of piperine on the oral absorption of linarin.

## 2. Results and Discussion

### 2.1. In Situ Permeability Study

P-gp is an efflux membrane transporter that can extrude a wide variety of endogenous and exogenous compounds from cells. It is well known that P-gp plays an important role in drug disposition and distribution. Drugs that are substrates of P-gp have the potential to be victims to drug-drug interactions. Prediction of P-gp substrate specificity can be estimated by the “rule of fours”. Compounds with (N + O) ≥ 8, MW > 400 and acid pK_a_ > 4 are likely to be P-gp substrates [20]. As for linarin, the number of O is 14 and the MW is 592 which indicate that linarin may be a substrate for P-gp. In the present study, an *in situ* perfusion model was used to estimate the effect of verapamil (P-gp inhibitor) on the intestinal absorption of linarin to confirm whether linarin is a substrate of P-gp. Meanwhile, the effect of piperine on the intestinal absorption of linarin was also investigated. Intestinal permeability of linarin was determined in rat jejunum segment using *in situ* single pass perfusion technique and the perfusate samples were analyzed by RP-HPLC. Effective permeability values were calculated from the steady-state drug concentrations in the perfusate collected from the outlet. The surgery and the single-pass perfusion were successfully performed in all rats included in the study. No loss of linarin was found when drug was perfused individually through the intestinal perfusion apparatus, indicating that there was no significant adsorption to the tubing. The analytes were found to be stable in the perfusion buffer and in the intestinal perfusate at 37 °C for at least 2 h (data not shown).

**Figure 2:**
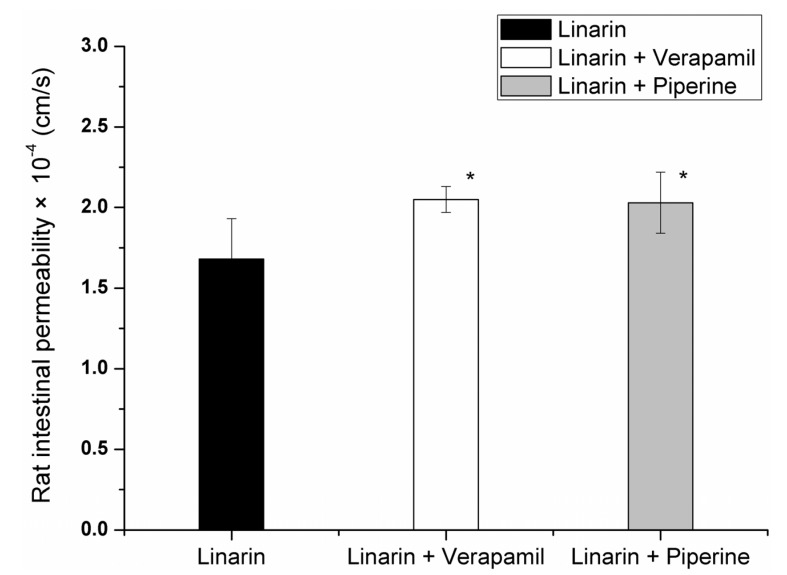
Rat intestinal permeability of linarin in the absence and presence of verapamil or piperine. * *p* < 0.05 as compared to linarin.

The net water flux values of linarin (10.8 μM), linarin co-perfused with verapamil (30 mM) or piperine (100 μM) were 0.69 ± 0.39, 0.80 ± 0.21 and 0.75 ± 0.46 μL/min/cm, respectively. It was found that the intestinal permeability coefficients (*P_eff_*) of linarin (10.8 μM) in the absence and presence of verapamil (30 mM) were (1.68 ± 0.25) × 10^−4^ and (2.05 ± 0.08) × 10^−4^ cm/s (*p* < 0.05), respectively. Linarin co-perfused with verapamil resulted in a significant increase in intestinal permeability (Figure 2). Accordingly, we speculated that linarin may be a substrate of P-gp, and efflux-mediated and saturable mechanisms might contribute to the poor intestinal absorption of linarin. Further studies were still needed to support this possibility. The *P_eff_* value of linarin upon co-perfusion with piperine (100 μM) was found to be (2.03 ± 0.19) × 10^−4^ cm/s (*p* < 0.05). Apparently, the presence of piperine increased the permeability of linarin significantly as compared to linarin alone (Figure 2). These results indicated that piperine could inhibit the function of P-gp in the intestine and modulate the intestinal absorption of linarin.

### 2.2. In Vivo Pharmacokinetic Study

**Figure 3:**
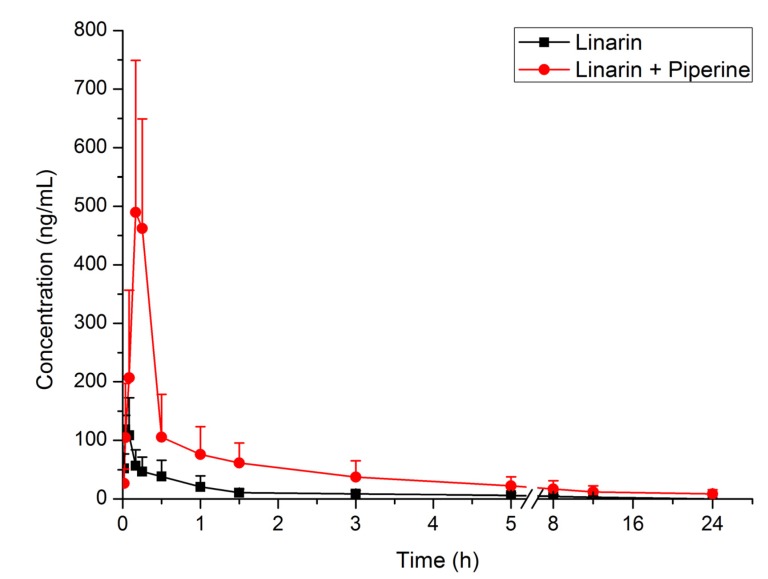
Mean plasma concentration–time curves of linarin in rats following oral administration of linarin alone and in combination with piperine (*n* = 5).

An *in vivo* pharmacokinetic model was used to estimate the effect of piperine on the oral absorption of linarin. The plasma concentrations of linarin at different time points after oral administration of linarin (50 mg/kg) alone and in combination with piperine (20 mg/kg) are listed in Table 1. The mean plasma concentration–time curves of linarin are shown in Figure 3. The main pharmacokinetic parameters of linarin are summarized in Table 2. It can be seen that co-administration of linarin with piperine resulted in significant changes in the pharmacokinetic properties of linarin. Compared to administration of linarin alone, the C_max_ and AUC of linarin were increased by 346% and 381%, respectively. The T_max_ was significantly delayed, whereas the CL/F was significantly decreased.

**Table 1:** Plasma concentrations of linarin at different time points following oral administration of linarin alone and in combination with piperine. (mean ± SD, *n* = 5).

Time (h)	Linarin	Linarin + Piperine
	(ng/mL)	(ng/mL)
0.017	52.12 ± 24.48	26.62 ± 24.10
0.033	118.91 ± 23.61	104.83 ± 92.52
0.083	108.81 ± 64.05	206.98 ± 149.73
0.167	56.60 ± 27.37	489.33 ± 259.63
0.25	46.66 ± 24.58	461.90 ± 187.20
0.5	38.20 ± 27.76	105.57 ± 73.11
1	20.40 ± 18.85	75.97 ± 47.60
1.5	10.46 ± 6.39	61.39 ± 34.09
3	8.44 ± 5.25	37.06 ± 27.87
5	5.99 ± 4.18	22.04 ± 15.70
8	4.22 ± 3.03	16.75 ± 14.30
12	3.13 ± 1.04	11.61 ± 10.61
24	0	8.32 ± 7.33

**Table 2:** Pharmacokinetic parameters of linarin following oral administration of linarin (50 mg/kg) alone and in combination with piperine (20 mg/kg) (mean ± SD, *n* = 5).

Parameters	Linarin	Linarin + Piperine
t_1/2_ (h)	4.2 ± 1.3	4.7 ± 2.8
CLz (L/h/kg)	556 ± 320	118 ± 75
AUC_0-t_ (ng·h/mL)	106 ± 67	554 ± 316
AUC_0-∞_ (ng·h/mL)	122 ± 76	587 ± 347
T_max_ (h)	0.053 ± 0.027	0.200 ± 0.046
C_max_ (ng/mL)	145 ± 32	647 ± 96

The enhanced oral exposure of linarin in the presence of piperine is partially due to the increased intestinal absorption of linarin via the inhibition of P-gp mediated linarin efflux, consistent with the results obtained in the *in situ* permeability study. In addition, the inhibition of the metabolism of linarin by piperine could also contribute to the significantly increased AUC and decreased CL/F of linarin. Several studies have revealed that piperine inhibits the metabolism of some compounds, such as curcumin and resveratrol [16,17]. Our previous study has demonstrated that linarin can be extensively metabolized in rats [21]. Therefore, the enhancement of oral absorption of linarin may be caused partially by the inhibition of the metabolism of linarin by piperine. Nevertheless, further study is still needed to support this possibility. Piperine was reported to affect tonic contractions of the proximal part of the stomach as well as the peristaltic activity of the antrum [13]. Therefore, the significant increase of the T_max_ of linarn in the presence of piperine may be explained by the delayed gastric emptying in the presence of piperine.

## 3. Experimental Section

### 3.1. Materials

Linarin (>98% purity) was obtained from Chengdu Must Bio-technology Co. Ltd. (Chengdu, China). Verapamil, piperine and baicalin (IS for LC–MS/MS assay) were all obtained from National Institute for the Control of Pharmaceutical and Biological Products (Beijing, China). HPLC-grade acetonitrile was purchased from Concord Technology Co. Ltd (Tianjin, China). All other reagents were of analytical grade. Doubly distilled water was used throughout the study.

### 3.2. Animals

Male Sprague-Dawley rats, 200–220 g, SPF, were provided by the Laboratory Animal Center of Shenyang Pharmaceutical University (Shenyang, China). All animals were housed under standard controlled environmental conditions: temperature (25 ± 2 °C), relative humidity (60% ± 10%), room air change (12–18 times/h) and light/dark cycle (12 h/12 h). Food and water were available ad libitum. The study was conducted under protocols approved by the Animal Ethics Committee of Shenyang Pharmaceutical University, in accordance with the Guide for the Care and Use of Laboratory Animals (NIH publication No.85-23, revised in 1985).

### 3.3. In Situ Intestinal Perfusion Experiment

The surgical procedure and *in situ* single-pass intestinal perfusion experiments were performed according to previously described methods [22,23]. After anesthesia via intraperitoneal administration of urethane (200 mg/kg), rats were placed on a heating pad to maintain body temperature at 37 °C. The abdomen was opened with a midline longitudinal incision. A 10–12 cm segment of the intestine (jejunum interface) was exposed and cannulated with plastic tubing (outer diameter, 4.77 mm; inner diameter, 3.17 mm). The intestinal segment was rinsed with isotonic saline (37 °C) until the outlet solution was clear. Perfusion buffer of 7–9 mL (NaCl, 7.8 g/L; KCl, 0.35 g/L; MgCl_2_, 0.02 g/L; CaCl_2_, 0.37 g/L; NaH_2_PO_4_, 0.32 g/L; NaHCO_3_, 1.37 g/L; glucose, 1.4 g/L) containing linarin (10.8 μM), with or without verapamil (30 mM) or piperine (100 μM), was allowed to equilibrate with the intestinal segment. Thereafter, the intestinal segment was perfused at a constant flow rate (Q) of 0.2 mL/min with a peristaltic pump. Each perfusion experiment lasted for 90 min, and perfusate was collected every 15 min. After the final sample was collected, the animals were euthanatized, the intestine was removed, and the length of the intestine was measured. All perfusate samples were weighed and then prepared for the HPLC assay. To 100 μL aliquot of perfusate sample, 100 μL of methanol was added. The mixture was then vortex-mixed for 1 min and centrifuged at 12,000 rpm for 5 min, and a 20 μL aliquot of the final solution was injected into the HPLC system for analysis.

Permeabilities were calculated after correcting the outlet concentration for water flux on the basis of the ratio of perfusion solution collected and infused for each sampling point (15 min).

The parameters below were calculated when the drug concentrations in the effluent perfusates were at steady state. The net water flux (NWF) was determined by a gravimetric method. *C_cor_*, the drug concentration of effluent perfusates which was corrected for water flux, was calculated according to the equation:


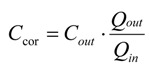
(1)

where *C_out_* is the concentration of tested drug in the effluent perfusates (μg/mL), *Q_in_* and *Q_out_* are the inlet and outlet flow rate, respectively, which are adjusted for liquid density (mL/min).

The effective permeability coefficients (*P_eff_*) were calculated from:


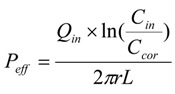
(2)

where *C_in_* is the concentration of tested drug in the influent perfusate; *2πrL* is the area of the mass transfer surface (cm^2^) within the intestinal segment which is assumed to be a cylinder area.

### 3.4. Pharmacokinetic Study

Rats were randomly divided into two groups (five rats per group). One group received a single dose of linarin (50 mg/kg) and the other group received linarin (50 mg/kg) and piperine (20 mg/kg) simultaneously. Linarin alone or with piperine were both dissolved in glycerol-alcohol-normal saline-dimethylacetamide (40:40:17:3, *v*/*v*/*v*/*v*) solution for oral administration.

Blood samples were collected from the eye retro-orbital sinus of rats into heparin-coated Eppendorf tubes at 0, 0.017, 0.033, 0.083, 0.17, 0.25, 0.50, 1.0, 1.5, 3.0, 5.0, 8.0, 12, 24 h after oral administration. The whole blood was immediately centrifuged at 12,000 rpm for 10 min. The obtained plasma was prepared with a liquid-liquid extraction procedure. To 20 μL aliquot of plasma sample, 20 μL of IS working solution was added, as well as 20 μL of methanol. The mixture was extracted with 600 μL of ethyl acetate by vortex-mixing for 1 min and shaking on an orbital shaker for 10 min. After centrifugation at 3500 rpm for 5 min, the upper organic phase was transferred into another tube and evaporated to dryness under a gentle stream of nitrogen at 37 °C. The residue was reconstituted in 50 μL of mobile phase and vortex-mixed for 30 s. A 5 μL aliquot of the final solution was injected into the LC–MS/MS system for analysis.

### 3.5. HPLC Assay

The HPLC system consisted of an Agilent 1100 series HPLC (Agilent Technologies, Palo Alto, CA, USA) with a quaternary pump and diode array detector (Agilent Technologies, Santa Clara, CA, USA). The separation was carried out on a Thermo ODS-2 HYPERSIL C_18_ column (200 mm × 4.6 mm I.D., 5 μm, Thermo Scientific, Pittsburgh, PA, USA) with a Diamonsil EasyGuard C_18_ guard cartridge (8 mm × 4 mm I.D., 5 μm, Dikma, Beijing, China) at 25 °C. The mobile phase consisted of acetonitrile−5 mM ammonium formate (35:65, *v*/*v*). The flow rate was set at 0.8 mL/min.

### 3.6. LC–MS/MS Assay

The LC–MS/MS system consisted of a Shimadzu SIL-HTA autosampler, a Shimadzu LC-10ADvp pump (Kyoto, Japan) and a Thermo Finnigan TSQ Quantum Ultra triple-quadrupole mass spectrometer (San Jose, CA, USA) equipped with an ESI interface. The separation was carried out on a Hypersil GOLD column (100 mm × 2.1 mm I.D., 5 μm, Thermo Scientific, Pittsburgh, PA, USA) with a Diamonsil EasyGuard C_18_ guard cartridge (8 mm × 4 mm I.D., 5 μm, Dikma, Beijing, China) at 20 °C. The mobile phase consisted of acetonitrile−2 mM ammonium formate (50:50, *v*/*v*). The flow rate was set at 0.2 mL/min. The ESI source was operated in positive ionization mode. The electrospray voltage was set at 4.2 kV and the capillary temperature was maintained at 320 °C. Nitrogen was used as the sheath gas (30 Arb) and auxiliary gas (5 Arb) for nebulization and desolvation. Argon was used as the collision gas (1.0 mTorr) for collision-induced dissociation. Selected reaction monitoring (SRM) was conducted by monitoring the precursor-product ion transitions of *m/z* 593→285 for linarin and *m/z* 447→271 for IS. The collision energies for linarin and IS were 30 eV and 15 eV, respectively. The sample injection volume was 5 µL. The calibration curves were linear over the concentration range of 0.001–1 μg/mL. The intra- and inter-day precisions in all samples were no more than 12.7%, while the accuracy was within ±5.2% of nominal values.

### 3.7. Data Analysis and Statistical Analysis

A non-compartmental model was used to calculate the pharmacokinetic parameters with DAS 2.1.1 software (Mathematical Pharmacology Professional Committee of China, Shanghai, China). Statistical comparison of mean values was performed by one-way analysis of variance (ANOVA). *p* < 0.05 was considered statistically significant.

## 4. Conclusions

In conclusion, the feasibility of improving the oral absorption of linarin in rats with a bioenhancer piperine was demonstrated for the first time. Piperine significantly increased the intestinal permeability of linarin. Co-administration of piperine increased the plasma exposure of linarin by 381% along with an increase in the C_max_ by 346%. These data are promising and form the basis for the further development and application of oral linarin. In future work, design and development of dual drug-loaded nanoformulations containing linarin and piperine, which may greatly increase the bioavailability of linarin, should be highly beneficial.
